# Rater agreement reliability of the dial test in the ACL-deficient knee

**DOI:** 10.1186/s40634-018-0131-y

**Published:** 2018-06-14

**Authors:** Malou E. Slichter, Nienke Wolterbeek, K. Gie Auw Yang, Jacco A. C. Zijl, Tom M. Piscaer

**Affiliations:** 1 Department of Orthopaedic Surgery, St. Antonius Hospital, P.O. Box 2500, 3430 EM Nieuwegein, The Netherlands; 2 Department of Orthopaedic Surgery, Erasmus Medical Center, P.O. Box 2040, 3000 CA Rotterdam, The Netherlands

**Keywords:** Dial test, Tibial external rotation, Rotatory laxity, Posterolateral rotatory instability, Anterior cruciate ligament, Posterior cruciate ligament, Posterolateral corner, Knee, Ligament

## Abstract

**Background:**

Posterolateral rotatory instability (PLRI) of the knee can easily be missed, because attention is paid to injury of the cruciate ligaments. If left untreated this clinical instability may persist after reconstruction of the cruciate ligaments and may put the graft at risk of failure. Even though the dial test is widely used to diagnose PLRI, no validity and reliability studies of the manual dial test are yet performed in patients. This study focuses on the reliability of the manual dial test by determining the rater agreement.

**Methods:**

Two independent examiners performed the dial test in knees of 52 patients after knee distorsion with a suspicion on ACL rupture. The dial test was performed in prone position in 30°, 60° and 90° of flexion of the knees. ≥10° side-to-side difference was considered a positive dial test. For quantification of the amount of rotation in degrees, a measuring device was used with a standardized 6 Nm force, using a digital torque adapter on a booth. The intra-rater, inter-rater and rater-device agreement were determined by calculating kappa (κ) for the dial test.

**Results:**

A positive dial test was found in 21.2% and 18.0% of the patients as assessed by a blinded examiner and orthopaedic surgeon respectively. Fair inter-rater agreement was found in 30° of flexion, κ_F_ = 0.29 (95% CI: 0.01 to 0.56), *p* = 0.044 and 90° of flexion, κ_F_ = 0.38 (95% CI: 0.10 to 0.66), *p* = 0.007. Almost perfect rater-device agreement was found in 30° of flexion, κ_C_ = 0.84 (95% CI: 0.52 to 1.15), *p* < 0.001. Moderate rater-device agreement was found in 30° and 90° combined, κ_C_ = 0.50 (95% CI: 0.13 to 0.86), *p* = 0.008. No significant intra-rater agreement was found.

**Conclusions:**

Rater agreement reliability of the manual dial test is questionable. It has a fair inter-rater agreement in 30° and 90° of flexion.

## Background

Posterolateral rotatory instability (PLRI) of the knee can easily be missed, because attention is paid to injury of the cruciate ligaments (Hughston & Jacobson, 1985; Kim et al., 2010). If left untreated this clinical instability may persist after reconstruction of the cruciate ligaments (O’Brien et al., 1991) and may put the graft at risk of failure (Harner et al., 2000; LaPrade et al., 2002; LaPrade et al., 1999). PLRI is defined as posterior subluxation and external rotation of the lateral tibial plateau in relation to the lateral femur condyle (Hughston et al., 1976; Hughston & Jacobson, 1985; Hughston & Norwood, 1980), as a consequence of injury to structures of the posterolateral corner (PLC) of the knee (Davies et al., 2004; Ferrari, 1999; Seebacher et al., 1982; Terry & LaPrade, 1994; Veltri & Warren, 1994). Multiple tests are described to diagnose PLRI, including the posterolateral drawer test (Hughston & Norwood, 1980), external rotation recurvatum test (Hughston & Norwood, 1980), reverse pivot shift test (Jakob et al., 1981), adduction stress test at 30° of flexion (Hughston & Jacobson, 1985) and an external rotation test (Loomer, 1991; Veltri & Warren, 1993), which is also known as the dial test or posterolateral rotation test (LaPrade & Wentorf, 2002; Larsen & Toth, 2005).

In 1991 Loomer (Loomer, 1991) described the dial test as a modification of the posterolateral drawer test. It is a tibial external rotation test based on biomechanical cutting studies (Gollehon et al., 1987; Grood et al., 1988) and has been extensively studied and described in the PCL-injured knee. Several ligament cutting studies (Bae et al., 2008; Grood et al., 1988; Strauss et al., 2007; Veltri et al., 1995; Wroble et al., 1993) and studies on clinical devices mimicking or measuring external rotation of the tibia, such as the dial test (Bae et al., 2008; Bleday et al., 1998; Forsythe et al., 2017; Krause et al., 2013; Lorbach et al., 2012; Musahl et al., 2007; Samukawa et al., 2007; Stinton et al., 2016; Tsai et al., 2008) have been performed. Its initial use was to distinguish between posterior cruciate ligament (PCL) injury solely and combined PCL and PLC injury. As is known, damage to the PLC may also be present in patients with anterior cruciate ligament (ACL) injury (Kim et al., 2010; LaPrade & Wentorf, 2002; O’Brien et al., 1991).

Many clinicians use the dial test in their daily practice for assessing PLRI in the ACL-deficient knee and whether or not to perform a PLC reconstruction of the knee at time of ACL reconstruction. The dial test is easy to perform and understand, and is widely accepted to diagnose PLRI of the knee in combination with other tests. We acknowledge the difficulty of relying on physical examination for the diagnosis of PLRI and no objective quantitative test exists for assessing increased external rotation. This makes accurate diagnosis of PLRI difficult (Laprade et al., 2014). As is known, a positive dial test may result from either posterolateral or medial knee injuries (Griffith et al., 2009; Wijdicks et al., 2010) and the results of rotatory instability tests should be carefully interpreted.

Literature on the reliability of the dial test is limited (Jung et al., 2009; Kim et al., 2010; Krause et al., 2013; Lee et al., 2015), especially in patients with ACL injury (Forsythe et al., 2017; Samukawa et al., 2007). At the moment no validity and reliability studies of the manual dial test are performed in patients with ACL injury yet.

Therefore, the purpose of this study is to determine the reliability of the manual dial test by focusing on the rater agreement in patients with suspected ACL injury and possible concomitant injury of the knee. We hypothesize that the dial test has good rater agreement.

## Methods

A monocenter study was performed to evaluate the rater agreement reliability of the dial test. This study was approved by the institutional review board of the author’s institution. All subjects provided written informed consent. The authors had no financial conflicts of interest.

Fifty-two patients between the ages of 18 and 50 years were included. Inclusion criteria were patients with a history of distorsion of the knee and thus strong suspicion on ACL injury or patients with proven ACL injury on magnetic resonance imaging (MRI) of the knee. Exclusion criteria included rheumatoid arthritis or other systemic inflammatory diseases, a history of previous injury or complaints of the contralateral knee, a locked knee, previous surgery on one of both knees, (congenital) malformation that could influence rotation of the knee or foot, asymmetrical rotation of the hips and asymmetrical leg axis.

### Procedure

The dial test was performed by two independent examiners as part of a full knee examination, including effusion, range of motion, anteroposterior stability tests, collateral stability test, rotatory instability tests and meniscal injury tests. All tests were scored according to the International Knee Document Committee (IKDC) criteria (Hefti et al., 1993). The first examiner was one of three orthopaedic knee surgeons, with ≥6 years of experience, participating in the study. The second examiner was a well-trained medical student blinded for the affected knee and was extensively trained on performing the physical examination of the knee by the senior orthopaedic surgeons. During a second follow-up appointment examination of the knees was repeated by the blinded examiner.

The dial test was performed in prone position with flexion angles of the knee of 30°, 60° and 90° (Veltri & Warren, 1993). Side-to-side difference of ≥10° of external rotation was considered a positive dial test. The test was scored either positive or negative.

### Quantification of external rotation in the knee

**Fig. 1:**
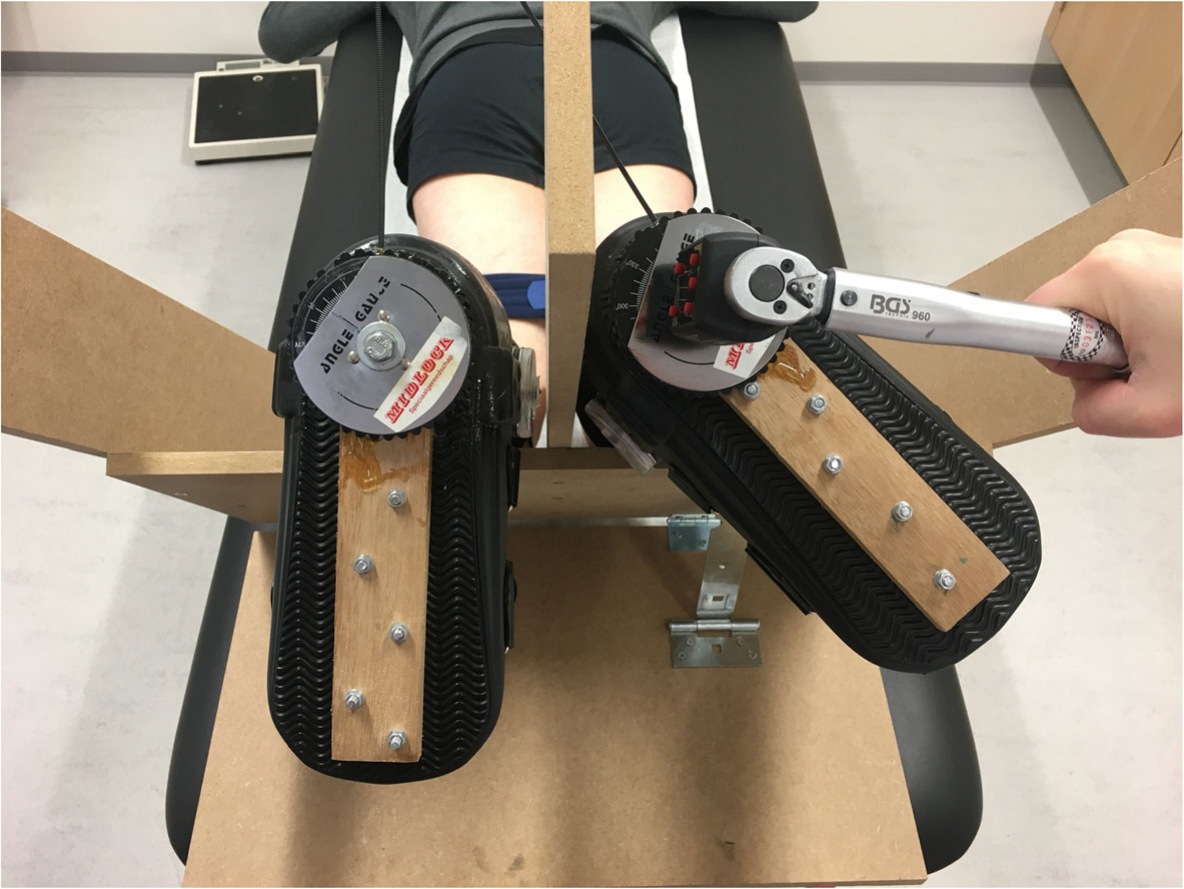
External rotation with 6 Nm torque

An objective quantification measuring device was developed and used for determining external rotation of the knee. The patient was placed in prone position with the knees in 30°, 60° and 90° of flexion. Support was provided for the legs while keeping accurate flexion angles of the knees and relative muscle relaxation was possible. Air inflatable walkers (protect.Air ROM Walker, size medium, Medi, Bayreuth, Germany) were used to fixate the ankle relative to the tibia to minimize the rotation of the ankle. It offers a snug fit, without being uncomfortable. A strap was used on both legs just proximal to the knees to avoid natural abduction of the hips. Based on biomechanical studies in vivo, a 6 Nm external torque was applied on both feet by means of a wrench with an electronic torque adapter (Kraftwerk Europe, art. 4081–14, ±2%) (Alam et al., 2013; Branch et al., 2010; Markolf et al., 1984; Mouton et al., 2012; Shultz et al., 2007; Tsai et al., 2008). With help of fixed angle gauges the thigh-foot angle was measured during external rotation of the ACL-insufficient knee and contralateral knee with both feet starting in neutral position and not the patient’s own resting position of the feet. Side-to-side difference of ≥10° after applying 6 Nm external torque was considered a positive dial test (Fig. 1).

### MRI of the injured knee

The MRI of the injured knee was assessed by a musculoskeletal radiologist as part of a routine protocol. All MRI’s were also assessed by the researcher for edema surrounding structures of the PLC, such as the LCL, popliteus tendon, popliteofibular ligament (PFL), biceps femoris tendon and the posterolateral capsule.

### Statistical analysis

Statistical analysis was performed using Statistical Package of the Social Sciences (SPSS, Chicago, IL, Version 24.0). Cohen’s kappa coefficients (κ_C_) were calculated to determine intra-rater agreement and rater-device agreement (Cohen, 1960). A Fleiss' kappa coefficient (κ_F_) was calculated for determining inter-rater agreement, because the two examiners are considered not unique (Fleiss, 1971). Rater number one was one of three participating orthopedic surgeons. The kappa values are typically interpreted as follows: <0.00, poor agreement; 0.00–0.20, slight agreement; 0.21–0.40, fair agreement; 0.41–0.60, moderate agreement; 0.61–0.80, substantial agreement; and 0.81–1.00, almost perfect agreement (Landis & Koch, 1977).

Literature on sample size estimation techniques is limited for observer agreement, therefore our aim was to include a minimum of 50 subjects (Donner, 1998; Donner & Eliasziw, 1992; Rotondi & Donner, 2012).

## Results

In this study, 57 patients were included, however, after physical examination 4 patients turned out to meet one of the exclusion criteria and were therefore excluded from the study. Another patient was examined only once by the researcher and was therefore excluded. Fifty (96%) patients were assessed for inter-rater agreement and 13 (25%) patients for intra-rater agreement. For rater-device agreement 19 (37%) patients were assessed of whom 7 were assessed again during second physical examination; therefore 26 pairs of knees were used. In 7 of the 52 patients (13%) the researcher was not blinded, because the patient accidentally gave out the affected side or was using crutches or an orthosis. For patients characteristics see Table 1.

**Table 1:** Patient characteristics of 52 patients

gender (men/women)	31 (59.6%)/21 (40.4%)
mean age in years ± SD	29.9 ± 9.2
mean body mass index in kg/m2 ± SD	24.4 ± 2.9
mean time between trauma and physical examination in weeks ± SD	23.4 ± 31.6
≤ 6 weeks	12 (23.1%)
> 6 weeks	39 (75%)
no recollection of trauma	1 (1.9%)
mean time between first physical examination and second physical examination in weeks ± SD	5.6 ± 3.8
mean time between trauma and MRI in weeks ± SD	16.4 ± 26.1
injured side (left/right)	20 (38.5%)/32 (61.5%)
unblinding of researcher	7 (13.5%)
inability of relative muscle relaxation	19 (36.5%)
physical therapy prior to first physical examination	37 (71.2%)
Treatment	
ACL reconstruction	29 (55.8%)
additional lateral extraarticular tenodesis^a^	4 (13.8%)
PLC reconstruction^b^	6 (20.7%)
conservative treatment	18 (34.6%)
diagnostic trajectory	5 (9.6%)

^a^Lemaire or modified Lemaire procedure

^b^Modified Larson procedure

All included patients showed increased anterior translation as assessed by the Lachmann test, anterior drawer test or pivot shift test when performed by either the orthopaedic surgeon or the blinded examiner. No increased posterior translation was found as assessed by a posterior drawer test.

In total, the blinded researcher found a positive dial test on the injured knee in 11 (21.2%) patients and found a positive test on the healthy knee in 4 (7.7%) patients during the first examination. The orthopedic surgeon found a positive test on the injured side in 9 (18.0%) patients and in 1 (2.0%) patient on the healthy knee. The overall proportion of agreement between raters was 70%. On the quantification measuring tool 8 (30.8%) of 26 patients had a positive test. Overall agreement between the researcher and the device was 62%.

Fair inter-rater agreement was found for the dial test in 30° and 90° flexion of the knees with κ_F_ = 0.29 (95% CI: 0.01 to 0.56), *p* = 0.044 and κ_F_ = 0.38 (95% CI: 0.10 to 0.66), *p* = 0.007 respectively (Table 2).

**Table 2:** Rater agreement of the dial test

	N=	κ	SE	95% confidence interval	*P* value
intra-rater (κ_C_)	13				
30° of flexion		0.16	0.28	−0.39–0.70	0.569
60° of flexion		0.35	0.27	−0.17–0.87	0.207
90° of flexion		0.49	0.25	0.01–0.98	0.071
30° and 90° combined		0.35	0.28	−0.19–0.89	0.207
inter-rater (κ_F_)	50				
30° of flexion		0.29	0.14	0.01–0.56	*0.044*
60° of flexion		0.22	0.14	−0.06–0.50	0.129
90° of flexion		0.38	0.14	0.10–0.66	*0.007*
30° and 90° combined		0.25	0.14	−0.03–0.53	0.077
rater-device (κ_C_)	26				
30° of flexion		0.84	0.16	0.52–1.15	*<0.001*
60° of flexion		0.20	0.23	−0.25–0.65	0.250
90° of flexion		0.26	0.23	−0.19–0.70	0.190
30° and 90° combined		0.50	0.19	0.13–0.86	*0.008*

No significant intra-rater agreement was found for the dial test with a Cohen’s kappa ranging from 0.16 to 0.49. An almost perfect rater-device agreement was found in 30° flexion of the knees, κ_C_ = 0.84 (95% CI: 0.52 to 1.15), *p* < 0.001. A moderate rater-device agreement was found in 30° and 90° combined, κ_C_ = 0.50 (95% CI: 0.13 to 0.86), *p* = 0.008.

From 16 patients with a positive dial test in 30°, 60° and/or 90°, 6 (37.5%) patients had an additional MCL lesion and another 6 (37.5%) patients had an additional LCL lesion. In this group a total of 6 (37.5%) patients showed abnormalities of the MCL or LCL on MRI (Table 3).

**Table 3:** Physical examination and MRI features indicating collateral injury in 52 patients with ACL injury with a positive and negative dial test

	positive dial test^a^ (*N* = 16)	negative dial test^a^ (*N* = 36)
varus gapping^a,b^		
grade A	10 (62.5%)	35 (97.2%)
grade B	4 (25%)	1 (2.8%)
grade C	2 (12.5%)	–
grade D	–	–
valgus gapping^a,b^		
grade A	10 (62.5%)	30 (8.3%)
grade B	4 (25%)	6 (16.7%)
grade C	2 (12.5%)	–
grade D	–	–
LCL on MRI		
intact	15 (93.8%)	31 (86.1%)
sprain	–	5 (13.9%)
partial rupture	1 (6.3%)	–
rupture	–	–
MCL on MRI		
intact	11 (68.8%)	29 (80.6%)
sprain	2 (12.5%)	5 (13.9%)
partial rupture	–	1 (2.8%)
rupture	3 (18.8%)	1 (2.8%)

^a^Assessed by either the blinded examiner and/or orthopaedic surgeon

^b^Grade A (normal; 0–2 mm), grade B (nearly normal; 3–5 mm), grade C (abnormal; 6–10 mm), grade D (severely abnormal; > 10 mm), according to the 2000 IKDC objective knee examination score

Seven patients were indicated for an additional PLC reconstruction at time of the ACL reconstruction based on physical examination performed by the orthopaedic surgeon and MRI features (Table 4). However, one patient turned out to have a negative dial test under anesthesia and no additional PLC reconstruction was performed.

**Table 4:** Physical examination^a^ and MRI features of 7 patients with ACL injury and an indication for PLC reconstruction

	dial 30°	dial 60°	dial 90°	varus gapping^b^	valgus gapping^b^	MRI
1	+	+	–	grade A	grade A	partial LCL rupture
2	+	+	–	grade A	grade A	no LCL or MCL injury
3	+	+	–	grade B	grade A	no LCL or MCL injury
4	+	–	–	grade B	grade A	no LCL or MCL injury
5	+	+	+	grade B	grade B	no LCL or MCL injury
6	+		+	grade C	grade B	no LCL or MCL injury
7^c^	+	+	–	grade B	grade A	no LCL or MCL injury

^a^Physical examination performed by the orthopaedic surgeon

^b^Grade A (normal; 0–2 mm), grade B (nearly normal; 3–5 mm), grade C (abnormal; 6–10 mm), grade D (severely abnormal; > 10 mm), according to the 2000 IKDC objective knee examination score

^c^No PLC reconstruction was performed

From 50 out of 52 (96%) patients the MRI of the ACL-injured knee was retrospectively reviewed, with mean time between trauma and MRI being 16.4 ± 26.1 weeks. From two patients the MRI was not assessable. Of all MRIs, 44 (88.0%) patients had ACL injury on MRI, 29 (58.0%) showed slight effusion surrounding the LCL, popliteus tendon, PFL or biceps femoris tendon. Other additional injury as seen on MRI is presented in Table 5.

**Table 5:** Features on MRI of the injured knee of 50 patients

ACL	
intact	4 (8%)
contusion	2 (4%)
partially ruptured	9 (18%)
ruptured	35 (70%)
PCL	
intact	46 (92%)
contusion	1 (2%)
buckling	3 (6%)
LCL	
intact	44 (88%)
sprain	5 (10%)
partial rupture	1 (2%)
MCL	
intact	38 (76%)
sprain	7 (14%)
partial rupture	1 (2%)
rupture	4 (8%)
intact popliteus tendon	50 (100%)
intact biceps tendon	50 (100%)
edema PLC^a^	29 (58%)
lesion of the lateral meniscus	13 (26%)
lesion of the medial meniscus	20 (40%)
bonebruise	
lateral femur condyl	30 (60%)
lateral tibiaplateau	35 (70%)
medial femur condyl	14 (28%)
medial tibiaplateau	8 (16%)

^a^Slight edema surrounding at least one structure of the PLC, such as the LCL, popliteus tendon, PFL, biceps femoris tendon and the posterolateral capsule

## Discussion

Structures of the PLC encompass the LCL, popliteus tendon and the popliteofibular ligament (Laprade et al., 2014). As is well-known, an injury of the LCL can clinically be differentiated from the popliteus tendon by performing both varus stress tests and the dial test (LaPrade et al., 2008; Laprade et al., 2010). Isolated LCL injury may result only in increased varus gapping and no increase in external rotation. On the other hand injury to the popliteus tendon may result in increased external rotation without varus gapping. Also, the heel height test has been described in the literature for the diagnosis of combined ACL and LCL tears (Cinque et al., 2017). Varus stress radiographs can provide an objective measure for diagnosis of LCL and posterolateral knee injuries (LaPrade et al., 2008).

In this study we focussed on increased external rotation as assessed by the dial test in the ACL-injured knee. The principal finding of this study is that the manual dial test has a fair inter-rater agreement in 30° and 90° of flexion in patients with ACL injury.

Reliability testing of the dial test has only been performed in several studies (Forsythe et al., 2017; Jung et al., 2009; Kim et al., 2010; Krause et al., 2013; Lee et al., 2015). These studies show substantial to almost perfect agreement, ranging from 0.60 to 0.92, when using an intraclass correlation coefficient (ICC) that can be used for quantitative measurements of tibial external rotation. However, the manual dial test is scored positive or negative by the clinician and no measurement tool is incorporated in to daily practice, therefore an ICC is not useful for determining reliability of the manual dial test. Results of these reliability studies (Forsythe et al., 2017; Jung et al., 2009; Kim et al., 2010; Krause et al., 2013; Lee et al., 2015) are difficult to compare as groups of patients, method of measurement and position in which the dial test is performed, differ from one another.

Likewise, significant correlations between the dial test and injury to structures of the PLC were found during surgical exploration (Kim et al., 2010; Laprade & Terry, 1997) and in selective ligament cutting studies performing external rotation of the tibia (Bae et al., 2008; Grood et al., 1988; Strauss et al., 2007; Veltri et al., 1995; Wroble et al., 1993). Nonetheless a cadaveric study of Bae et al. (Bae et al., 2008) suggests that in patients with ACL injury, injury to less than three posterolateral structures (excluding the PCL) might not be clinically detected by the dial test. Also Veltri et al. (Veltri et al., 1995) state that the standard external rotation test performed at 30° of knee flexion may not be routinely reliable for detecting combined anterior cruciate and posterolateral ligament injury. They suggest that increased total rotation may be indicative for injury to the posterolateral structures in the ACL-deficient knee. Furthermore, a study of Griffith et al. (Griffith et al., 2009) shows that significant increases in external rotation at 30° of knee flexion were found with all medial knee structures sectioned, which indicates that a positive dial test may be found not only for posterolateral knee injuries but also for medial knee injuries. Therefore, careful correlation with the results of valgus stress testing and assessment of the location of tibial subluxation during the dial test, performed in prone and supine position, is necessary to distinct between possible posterolateral or medial knee injury (Griffith et al., 2009; Wijdicks et al., 2010). Also comparing the lateral tibial condyle with the medial tibial condyle during posterolateral and anteromedial drawer tests can aid in this distinction (Hughston & Norwood, 1980; Wijdicks et al., 2010).

There is no consensus in literature whether the dial test should be performed in prone or supine position in the ACL-deficient knee (Lunden et al., 2010). It was first described in supine position (Loomer, 1991) and later on in prone position (Veltri & Warren, 1993). Performing the dial test in prone position is considered the same as applying an anterior force to the tibia when performed in supine position (Jung et al., 2009; Strauss et al., 2007). Even though no posterior tibial subluxation will be visible if the posterior subluxation is reduced in the PCL-injured knee, it increases the ability of an examiner to detect a concomitant PLC injury in the setting of a PCL-deficient knee (Jung et al., 2009; Lee et al., 2015; Strauss et al., 2007). No such studies are performed in the ACL-deficient knee. Therefore, we chose to perform the dial test in prone position in the suspected ACL-injured patient, because it offers easy control of the flexion angles in the knee, flexion of the hips, manual torque applied and requires only one examiner.

The dial test is considered positive if ≥10° side-to-side difference is present (Veltri & Warren, 1993), however as is known side-to-side differences are present in healthy subjects and in the ACL-injured knee without concomitant PLC injury (Forsythe et al., 2017; Krause et al., 2013). A study from Krause et al. (Krause et al., 2013) demonstrated side-to-side differences ranging from 4.7° to 5.9° in healthy subjects. They show that a threshold of ≥10° side-to-side difference results in incorrect positive testing in 21% of their subjects and therefore suggest a threshold of ≥15° side-to-side difference. Even though biomechanical cadaveric studies suggest that the ACL does play a secondary role in restraining internal tibial rotation (Mouton et al., 2012; Wroble et al., 1993), a study of Forsythe et al. (Forsythe et al., 2017) demonstrated that isolated deficiency of the ACL accounts for nearly 7° of tibial external rotation found by the dial test in the absence of PLC injury and caution should be taken when interpreting the dial test.

Besides variations in applied torque by different examiners, the amount of laxity can also influence the result of the dial test. Cooper et al. (Cooper, 1991) performed the dial test on healthy subjects under anesthesia. They found that the amount of external rotation laxity varied inversely according to their laxity index and that the thigh-foot angle only indirectly correlates with tibial rotation at the knee. Another study of Stinton et al. (Stinton et al., 2016) shows contradictory results. They state that the foot is not an accurate measure of tibial rotation and that the dial test performed by the clinician should focus on observation at the tibial tubercle and not on the foot. Because of these factors affecting the result of the dial test, it might be beneficial that an increase of side-to-side difference of external tibial rotation must be evaluated as a part of maximum external rotation, resulting in a percentage. This eliminates the individual variety in rotation laxity.

In summary, several patients with ACL injury showed increased external rotation of the knee as assessed by the manual dial test. This study found fair inter-rater agreement of the manual dial test performed in prone position in patients with ACL injury. Based on this study rater agreement reliability of the manual dial test is questionable in the patient suspected for ACL injury, with or without concomitant injury. Diagnosing PLRI depends highly on the orthopaedic surgeon performing the dial test if no injury to the PLC is seen on MRI. This can result in high variety in the decision of whether or not to perform an additional PLC reconstruction amongst orthopaedic surgeons. Caution should be taken when addressing PLRI with a PLC reconstruction if solely based on a positive dial test. Therefore other tests should be performed to eliminate other causes of increased external rotation in the ACL-injured knee, such as concomitant medial injury. Ideally, the results of this study should be compared to a healthy control group for further clarification on this matter. We believe that the dial test provides valuable information about the external rotation laxity of the knee. Also, further research should focus on quantification devices of tibial rotation (Bae et al., 2008; Bleday et al., 1998; Jung et al., 2009; Krause et al., 2013; Lorbach et al., 2012; Musahl et al., 2007; Stinton et al., 2016; Tsai et al., 2008), so that the increase of tibial rotation can be evaluated in relation to total rotation.

This study has several limitations. We did not compare results of the manual dial test performed in prone position compared to the supine position. Also, we did not differentiate between injury to the PLC or the medial structures which could both result in a positive dial test. No ICC was calculated for the manual dial test due to dichotomy of the results. Therefore, no minimal detectable change (MDC) and minimal clinically important difference (MCID) were calculated. We tried to minimize factors that influence the result of the dial test. However, the accuracy of the test depends on applied torque of the examiner, accuracy of estimating 10° side-to-side difference and laxity of the patient. Moreover, the patient’s reflex to resist instability tests due to discomfort or fear may result in some inaccuracies, such as a positive dial test on the healthy contralateral knee. Also, the orthopaedic surgeon was not blinded in contrast to the second examiner. No significant intra-rater agreement was found, possibly due to a large time interval between the assessments and a small sample size, resulting in large confidence intervals. Also, we do not know whether the time between ACL injury and physical examination influences the amount of external rotation and thus the outcome of the dial test. Because of the absence of a gold standard and the heterogeneity of concomitant injury of our patient population no sensitivity and specificity for the dial test could be calculated for diagnosing PLC injury. However, we do believe this is a representative and clinically relevant group of patients in whom the dial test should be performed.

## Conclusion

According to our study the manual dial test performed in prone position has a fair inter-rater agreement in 30° and 90° of flexion in patients with ACL injury. Therefore its rater agreement reliability is questionable in the ACL-injured knee and its findings should be interpreted with caution.

## Abbreviations

ACL: Anterior cruciate ligament
ICC: Intraclass correlation coefficent
IKDC: International Knee Document Committee
LCL: Lateral collateral ligament
MCID: Minimal clinically important difference
MCL: Medial collateral ligament
MDC: Minimal detectable change
MRI: Magnetic resonance imaging
Nm: Newtonmeter
PCL: Posterior cruciate ligament
PFL: Popliteofibular ligament
PLC: Posterolateral corner
PLRI: Posterolateral rotatory instability
SE: Standard error
SPSS: Statistical Package of the Social Sciences
κ_C_: Cohen’s kappa
κ_F_: Fleiss’ kappa

## References

[CR1] Alam M, Bull AMJ, deW TR, Amis AA (2013). A clinical device for measuring internal-external rotational laxity of the knee. Am J Sports Med.

[CR2] Bae JH, Choi IC, Suh SW, Lim HC, Bae TS, Nha KW, Wang JH (2008). Evaluation of the reliability of the dial test for posterolateral rotatory instability: a cadaveric study using an isotonic rotation machine. Arthroscopy.

[CR3] Bleday RM, Fanelli GC, Giannotti BF, Edson CJ, Barrett TA (1998). Instrumented measurement of the posterolateral corner. Arthroscopy.

[CR4] Branch TP, Browne JE, Campbell JD, Siebold R, Freedberg HI, Arendt EA, Lavoie F, Neyret P, Jacobs CA (2010). Rotational laxity greater in patients with contralateral anterior cruciate ligament injury than healthy volunteers. Knee Surg Sports Traumatol Arthrosc.

[CR5] Cinque ME, Geeslin AG, Chahla J, Moatshe G, Pogorzelski J, DePhillipo NN, LaPrade RF (2017). The heel height test: a novel tool for the detection of combined anterior cruciate ligament and fibular collateral ligament tears. ArthroscopyJournal of Arthroscopic and Related Surgery.

[CR6] Cohen J (1960). A coefficient of agreement for nominal scales. Educ Psychol Meas.

[CR7] Cooper DE (1991). Tests for posterolateral instability of the knee in normal subjects. Results of examination under anesthesia. J Bone Jt Surg Am.

[CR8] Davies H, Unwin A, Aichroth P (2004). The posterolateral corner of the knee. Anatomy, biomechanics and management of injuries. Injury.

[CR9] Donner A (1998). Sample size requirements for the comparison of two or more coefficients of inter-observer agreement. Stat Med.

[CR10] Donner A, Eliasziw M (1992). A goodness-of-fit approach to inference procedures for the kappa statistic: confidence interval construction, significance-testing and sample size estimation. Stat Med.

[CR11] Ferrari JD, Bach Jr. BR (1999) Posterolateral instability of the knee: diagnosis and treatment of acute and chronic instability. Sports Med Arthrosc 7:273–288

[CR12] Fleiss J (1971). Measuring nominal scale agreement among many raters. Psychol Bull.

[CR13] Forsythe B, Saltzman BM, Cvetanovich GL, Collins MJ, Arns TA, Verma NN, Cole BJ, Bach Jr. BR (2017) Dial test: unrecognized predictor of anterior cruciate ligament deficiency. Arthroscopy 33:1375–1381 10.1016/j.arthro.2017.01.043 28343807

[CR14] Gollehon DL, Torzilli PA, Warren RF (1987). The role of the posterolateral and cruciate ligaments in the stability of the human knee. A biomechanical study. J Bone Jt Surg Am.

[CR15] Griffith CJ, LaPrade RF, Johansen S, Armitage B, Wijdicks C, Engebretsen L (2009). Medial knee injury: part 1, static function of the individual components of the main medial knee structures. Am J Sports Med.

[CR16] Grood ES, Stowers SF, Noyes FR (1988). Limits of movement in the human knee. Effect of sectioning the posterior cruciate ligament and posterolateral structures. J Bone Jt Surg Am.

[CR17] Harner CD, Vogrin TM, Höher J, Ma CB, Woo SL (2000). Biomechanical analysis of a posterior cruciate ligament reconstruction. Deficiency of the posterolateral structures as a cause of graft failure. Am J Sports Med.

[CR18] Hefti E, Müller W, Jakob RP, Stäubli HU (1993). Evaluation of knee ligament injuries with the IKDC form. Knee Surg Sports Traumatol Arthrosc.

[CR19] Hughston JC, Andrews JR, Cross MJ, Moschi A (1976). Classification of knee ligament instabilities part II. The lateral compartment. J Bone Jt Surg Am.

[CR20] Hughston JC, Jacobson KE (1985). Chronic posterolateral rotatory instability of the knee. J Bone Jt Surg Am.

[CR21] Hughston JC, Norwood LA (1980). The posterolateral drawer test and external rotational recurvatum test for posterolateral rotatory instability of the knee. Clin Orthop Relat Res.

[CR22] Jakob RP, Hassler H, Staeubli HU (1981). Observations on rotatory instability of the lateral compartment of the knee: experimental studies on the functional anatomy and the pathomechanism of the true and the reversed pivot shift sign. Acta Orthop Scand.

[CR23] Jung YB, Lee YS, Jung HJ, Nam CH (2009). Evaluation of posterolateral rotatory knee instability using the dial test according to tibial positioning. Arthroscopy.

[CR24] Kim JG, Lee YS, Kim YJ, Shim JC, Ha JK, Park HA, Yang SJ, Oh SJ (2010). Correlation between the rotational degree of the dial test and arthroscopic and physical findings in posterolateral rotatory instability. Knee Surg Sports Traumatol Arthrosc.

[CR25] Krause DA, Levy BA, Shah JP, Stuart MJ, Hollman JH, Dahm DL (2013). Reliability of the dial test using a handheld inclinometer. Knee Surg Sports Traumatol Arthrosc.

[CR26] Landis JR, Koch GG (1977). The measurement of observer agreement for categorical data. Biometrics.

[CR27] Laprade RF, Griffith CJ, Coobs BR, Geeslin AG, Johansen S, Engebretsen L (2014). Improving outcomes for posterolateral knee injuries. J Orthop Res.

[CR28] LaPrade RF, Heikes C, Bakker AJ, Jakobsen RB (2008). The reproducibility and repeatability of varus stress radiographs in the assessment of isolated fibular collateral ligament and grade-III posterolateral knee injuries. An in vitro biomechanical study. J Bone Jt Surg Am.

[CR29] LaPrade RF, Muench C, Wentorf F, Lewis JL (2002). The effect of injury to the posterolateral structures of the knee on force in a posterior cruciate ligament graft. A biomechanical study Am J Sports Med.

[CR30] LaPrade RF, Resig S, Wentorf F, Lewis JL (1999). The effects of grade III posterolateral knee complex injuries on anterior cruciate ligament graft force. A biomechanical analysis Am J Sports Med.

[CR31] Laprade RF, Terry GC (1997). Injuries to the posterolateral aspect of the knee. Association of anatomic injury patterns with clinical instability. Am J Sports Med.

[CR32] LaPrade RF, Wentorf F (2002). Diagnosis and treatment of posterolateral knee injuries. Clin Orthop Relat Res.

[CR33] Laprade RF, Wozniczka JK, Stellmaker MP, Wijdicks CA (2010). Analysis of the static function of the popliteus tendon and evaluation of an anatomic reconstruction: the “fifth ligament” of the knee. Am J Sports Med.

[CR34] Larsen MW, Toth A (2005). Examination of posterolateral corner injuries. J Knee Surg.

[CR35] Lee HJ, Park YB, Ko YB, Kim SH, Bin KH, Yu DS, Jung YB (2015). The necessity of clinical application of tibial reduction for detection of underestimated posterolateral rotatory instability in combined posterior cruciate ligament and posterolateral corner deficient knee. Knee Surg Sports Traumatol Arthrosc.

[CR36] Loomer RL (1991). A test for knee posterolateral rotatory instability. Clin Orthop Relat Res.

[CR37] Lorbach O, Brockmeyer M, Kieb M, Zerbe T, Pape D, Seil R (2012). Objective measurement devices to assess static rotational knee laxity: focus on the rotameter. Knee Surg Sports Traumatol Arthrosc.

[CR38] Lunden JB, Bzdusek PJ, Monson JK, Malcomson KW, Laprade RF (2010). Current concepts in the recognition and treatment of posterolateral corner injuries of the knee. J Orthop Sports Phys Ther.

[CR39] Markolf KL, Kochan A, Amstutz HC (1984). Measurement of knee stiffness and laxity in patients with documented absence of the anterior cruciate ligament. J Bone Jt Surg Am.

[CR40] Mouton C, Theisen D, Pape D, Nührenbörger C, Seil R (2012). Static rotational knee laxity in anterior cruciate ligament injuries. Knee Surg Sports Traumatol Arthrosc.

[CR41] Musahl V, Bell KM, Tsai AG, Costic RS, Allaire R, Zantop T, Irrgang JJ, Fu FH (2007). Development of a simple device for measurement of rotational knee laxity. Knee Surg Sports Traumatol Arthrosc.

[CR42] O’Brien SJ, Warren RF, Pavlov H, Panariello R, Wickiewicz TL (1991). Reconstruction of the chronically insufficient anterior cruciate ligament with the central third of the patellar ligament. J Bone Jt Surg Am.

[CR43] Rotondi MA, Donner A (2012). A confidence interval approach to sample size estimation for interobserver agreement studies with multiple raters and outcomes. J Clin Epidemiol.

[CR44] Samukawa M, Magee D, Katayose M (2007). The effect of tibial rotation on the presence of instability in the anterior cruciate ligament deficient knee. J Sport Rehabil.

[CR45] Seebacher J, Inglis A, Marshall J, Warren R (1982). The structure of the posterolateral aspect of the knee. J Bone Jt Surg Am.

[CR46] Shultz SJ, Shimokochi Y, Nguyen AD, Schmitz RJ, Beynnon BD, Perrin DH (2007). Measurement of varus-valgus and internal-external rotational knee laxities in vivo - part I: assessment of measurement reliability and bilateral asymmetry. J Orthop Res.

[CR47] Stinton SK, Siebold R, Freedberg H, Jacobs C, Branch TP (2016). The use of a robotic tibial rotation device and an electromagnetic tracking system to accurately reproduce the clinical dial test. Knee Surg Sports Traumatol Arthrosc.

[CR48] Strauss EJ, Ishak C, Inzerillo C, Walsh M, Yildirim G, Walker P, Jazrawi L, Rosen J (2007). Effect of tibial positioning on the diagnosis of posterolateral rotatory instability in the posterior cruciate ligament-deficient knee. Br J Sports Med.

[CR49] Terry GC, LaPrade RF (1994). The posterolateral aspect of the knee. Anatomy and surgical approach. Am J Sports Med.

[CR50] Tsai AG, Musahl V, Steckel H, Bell KM, Zantop T, Irrgang JJ, Fu FH (2008) Rotational knee laxity: reliability of a simple measurement device in vivo. BMC Musculoskelet Disord. 10.1186/1471-2474-9-35 10.1186/1471-2474-9-35 PMC2315651 18366671

[CR51] Veltri DM, Deng X-H, Torzilli PA, Warren RF, Maynard MJ (1995). The role of the cruciate and posterolateral ligaments in stability of the knee. A biomechanical study. Am J Sports Med.

[CR52] Veltri DM, Warren RF (1993). Isolated and combined posterior cruciate ligament injuries. J Am Acad Orthop Surg.

[CR53] Veltri DM, Warren RF (1994). Posterolateral instability of the knee. J Bone Jt Surg Am.

[CR54] Wijdicks CA, Griffith CJ, Johansen S, Engebretsen L, LaPrade RF (2010). Injuries to the medial collateral ligament and associated medial structures of the knee. J Bone Jt Surg Am.

[CR55] Wroble RR, Grood ES, Cummings JS, Henderson JM, Noyes FR (1993). The role of the lateral extraarticular restraints in the anterior cruciate ligament-deficient knee. Am J Sports Med.

